# The Current State and Needs of North American Movement Disorders Fellowship Programs

**DOI:** 10.1155/2013/701426

**Published:** 2013-07-25

**Authors:** Ludy C. Shih, Daniel Tarsy, Michael S. Okun

**Affiliations:** ^1^Department of Neurology, Beth Israel Deaconess Medical Center, Kirstein 228, 330 Brookline Avenue, Boston, MA 02215, USA; ^2^Department of Neurology, University of Florida Center for Movement Disorders and Neurorestoration, Gainesville, FL 32607, USA

## Abstract

*Background*. Movement disorders fellowships are an important source of future clinician-specialists and clinician-scientists for the field. Scant published information exists on the number and characteristics of North American movement disorders fellowship training programs. *Methods*. A 31-item internet-based survey was formulated and distributed to academic movement disorders listed in the American Academy of Neurology (AAN) directory as having a movement disorders fellowship and to all National Parkinson Foundation Centers of Excellence and Care Centers in North America. *Results*. There was a 77% response rate among academic movement disorders centers. Broad similarities in clinical training were identified. The two most important rated missions of maintaining a movement disorders fellowship were contributions to scholarly activities and to fulfilling a critical need for specialists. Almost a quarter of fellowship programs did not offer a fellowship slot during the most recent academic year. Fellowship directors cited a wide variety of funding sources, but their top concern was lack of available funding for fellowship programs. *Conclusions*. North American movement disorders fellowship training programs currently offer similar methods of clinical training and education. Lack of funding was the most important obstacle to maintaining fellowship programs and should be made a priority for discussion in the field.

## 1. Introduction

Movement disorders fellowship training programs in North America are postresidency training experiences that are specifically designed to provide neurologists with expertise in the diagnosis and management of conditions such as Parkinson's disease (PD), parkinsonism, tremor, dystonia, tics, chorea, myoclonus, and other basal ganglia-related movement disorders. Since the 1980s in North America, there has been a steady growth from a handful of centers to the current landscape which includes dozens of centers that offer subspecialty training in movement disorders. Little published data exists concerning the type of training, the curriculum content, and the clinical experience that is offered by these training programs. Only one publication has detailed the clinical training and didactic experience at a single large movement disorders center and fellowship training program [1].

United States-based programs are not funded by the government or American Council on Graduate Medical Education (ACGME). Concern exists among fellowship directors that available support and training will not meet the needs of an aging population. Additionally, the literature has suggested that meaningful differences exist between movement disorders specialist care and general neurologist care [2–5]. We sought to gather information from fellowship program directors on training characteristics, educational programs, funding sources, and current challenges to maintaining the movement disorders subspecialty training experience.

## 2. Methods

We conducted an online survey (http://www.surveymonkey.com/) with the approval of the Institutional Review Board at the Beth Israel Deaconess Medical Center. Names of National Parkinson Foundation (NPF) Centers of Excellence and Care Center Directors as well as names of fellowship program directors listed in the AAN fellowship directory were compiled. A list of 87 programs was compiled, including 62 academic movement disorders centers and an additional 25 designated NPF Centers of Excellence or NPF Care Centers. The original survey was sent in March 2012 by email link (see Supplemental Material available online at http://dx.doi.org/10.1155/2013/701426) to fellowship directors with specific instructions for only one person to reply if two separate directors were listed. The email survey link was redistributed four weeks and eight weeks after the initial notice. Data analysis was performed using http://www.surveymonkey.com/ and Excel. All responders were asked to identify their program by geographic location, name (optional), special center affiliations (American Parkinson's Disease Association (APDA) Center of Excellence, NPF Center of Excellence, etc.), and whether they currently offered a movement disorders fellowship training program. If the response to the latter question was affirmative, respondents were asked to complete the 31-item survey. Throughout the survey, freeform text response options were made available for any comments not addressed by the answer options provided.

## 3. Results

### 3.1. Movement Disorders Centers Characteristics

**Figure 1:**
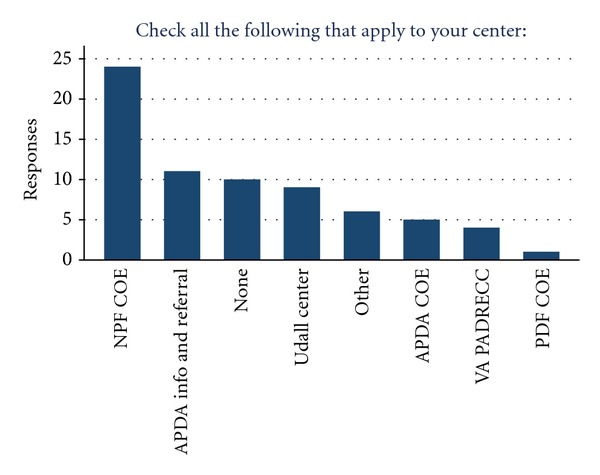
Special designations with respect to research or clinical care.

More than half of the 87 centers (*n* = 51, 58.6%) responded. Slightly more than three quarters (48/62, or 77.4%) of 62 academic movement disorders centers responded. Responders represented 23 states plus the District of Columbia and four Canadian provinces. A wide variety of center designations were reported, with 24 programs reporting NPF Center of Excellence status, and 11 programs reporting APDA Information and Referral Center status. Some centers also reported status as an NIH Udall Parkinson's Center (*n* = 9), APDA Center of Excellence (*n* = 5), Veterans Affairs Parkinson's Disease Research Education and Clinical Center (VA PADRECC) (*n* = 4), and Parkinson's Disease Foundation (PDF) Center of Excellence *n* = 1 (Figure 1). 

All but four centers (*n* = 47, 92%) stated that they offered a fellowship program, and those with a fellowship program were invited to continue the survey. Of the four centers that indicated that they were not offering fellowship programs, three stated that there were “not enough funds to cover fellow's salary,” and one stated that there was “too much administrative work.” A “lack of affiliation with an academic or university training program” was cited twice.

### 3.2. Number of Fellowship Training Slots

The number of fellowship training positions each year varied among institutions. Even among programs who responded that they currently offered fellowship training, 10 programs did not offer fellowship slots in the 2011-12 academic year. Overall, for the most 2011-12 academic year, 33 out of 43 (76.7%) programs responded that they started at least one new fellow. Slightly more than half of the programs responded that one fellowship position had commenced that year (*n* = 23, 53.5%), seven programs started two fellows (16.3%), and three programs started three fellows (7.0%), for a total of 46 fellows who started training in the 2011-12 academic year. The percentage of programs who started at least one new fellow (76.7%) was smaller compared to the preceding four academic years 2006–2011, 82.9% (*n* = 41), 82.5% (*n* = 40), 86.8% (*n* = 38), and 83.3% (*n* = 36). Most programs (86%) felt “more than 95% likely” that they would be offering at least one fellowship position in the academic year 2012-13, which was to begin three months following survey collection.

Fellowship directors were queried as to whether they restricted fellowship slots to domestic neurology residency program graduates. Only 24.4% of responders offered fellowships to only domestic medical graduates. Program directors estimated that 26.4% of their fellowship slots were filled by foreign medical graduates.

### 3.3. Duration, Structure, and Content of the Training Program

#### 3.3.1. Duration of Training

The duration of training fell predominantly into one of two categories. There were 18 programs (40%) who reported that the duration of training was a 2 year minimum, and funding for both years was available. Another 18 programs (40%) responded that one year was the minimum duration, but a second year was possible, if institutional funding was available. The remaining 20% of programs responded that the duration of their program was one year only, with no option for continuing (*n* = 5, 11.1%), or one year minimum, with the option for the 2nd year of training contingent upon the fellow's ability to successfully obtain his or her own grant funding for the second year (*n* = 3, 6.7%).

#### 3.3.2. Structure of Training and Clinical Exposure

Responders were also queried about the content of the training for outpatient clinical experiences, clinical research, teaching, and other scholarly activities. When asked how many half-day clinic sessions per week first year fellows spent in ambulatory clinic, 40% of programs reported six sessions weekly, and 20% reported seven or more clinic sessions, while another 8.9% reported five sessions weekly. All programs responded that fellows did some clinical research, with 62% reporting either one or two half-days per week, while another 31% reported three to five half-days. Three programs were research intensive, reporting seven or more half-days devoted to either clinical or basic research. 

Most programs reported that first year fellows performed some inpatient consultations, either one or less than one half-day per week (72.7%). Additionally, most programs, 83.7%, reported that one or less than one half-day was used for teaching and education of residents and medical students. Three-quarters of the programs reported that one or less than one half-day was used for formal didactic classes. 

Slightly more than half of the fellowship programs (*n* = 25) responded to questions about the second year schedule. The second year was similar to the first year schedule in all respects with exception of the distribution of time spent between ambulatory clinic and clinical research. The number of ambulatory clinic sessions was more variable, with 37% reporting four sessions weekly, 22.2% reporting five sessions weekly, and only 3.7% reporting six sessions weekly. Time devoted to clinical research increased, with 62% of programs reporting three to five half-days spent on this activity.

We also surveyed the approximate percentage of diagnoses and conditions comprising the fellow's clinical exposure. The average response reported Parkinson's disease (PD) 51.9% (range 20–80%), dystonia 15.1% (range 4–25%), tremor disorders 13.7% (range 2–30%), ataxia 7.5% (range 2–20%), and other movement disorders such as tics, tardive syndromes, and chorea 10.7% (range 1–30%). Only seven programs responded that at least some nonmovement disorders general neurology patients were seen, although in these cases they were generally low proportions (range 3–10%).

A vast majority of programs indicated that dedicated clinics were set up for botulinum toxin injections (97.8%), deep brain stimulation (DBS) programming (82.2%), and DBS intraoperative sessions (62.2%). Many programs indicated dedicated clinic sessions to PD, Huntington's disease, ataxia, atypical parkinsonism, dystonia, tremors, and tic disorders, but most programs (73.3%) also indicated that these conditions were also seen throughout the general movement disorders clinics. One program each also indicated that they had a dedicated neurogenetics clinic and a developmental disabilities clinic.

#### 3.3.3. Curriculum Content and Conferences

**Figure 2:**
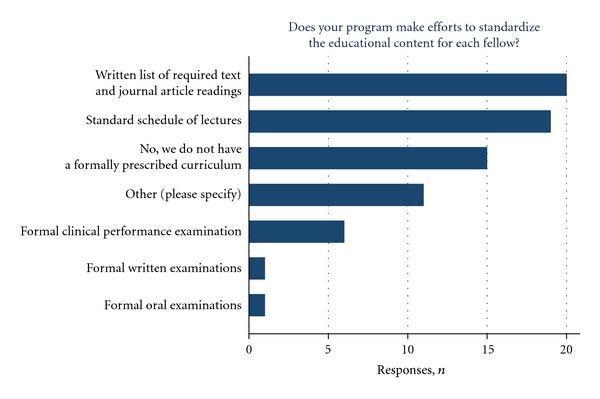
Responders describe efforts at standardizing educational content for each fellow.

**Figure 3:**
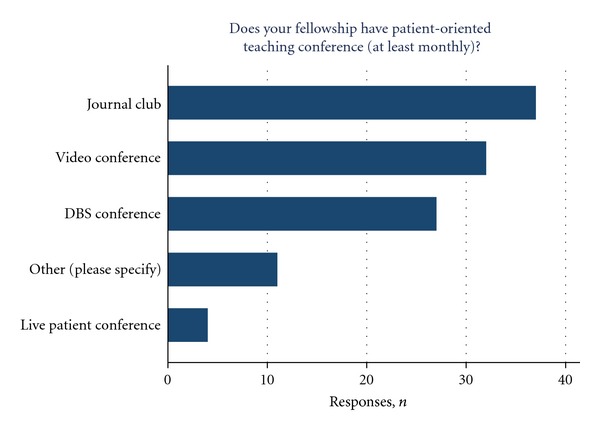
Number of programs that hold regular patient oriented teaching conferences.

Efforts at standardizing fellowship curriculum and scheduling scholarly activities were present at the vast majority of fellowship programs. Nearly half of the programs reported a written list of required text and journal article readings (44.4%), and a standard schedule of lectures covering specific topics aimed towards the fellows educational experience (42.2%, Figure 2). DBS conference, journal club, and video conference were present in the majority of the programs (Figure 3).

### 3.4. Faculty Supervision, Accreditation, and Funding Sources

**Figure 4:**
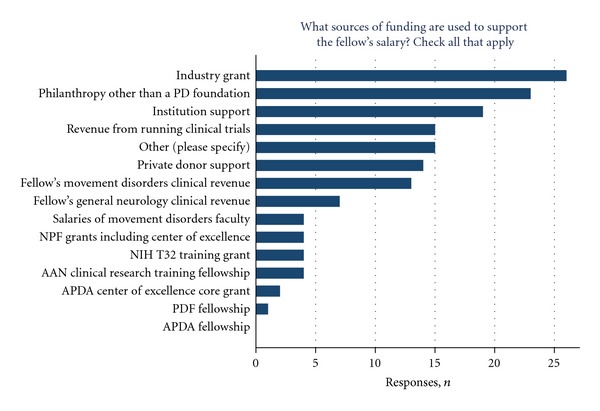
Sources of funding for fellowship support. Other responses include Parkinson Society of Canada, Dystonia Medical Research Foundation, NIH intramural funds, PADRECC grant, VA grant, and faculty NIH extramural grant.

The average number of faculty affiliated with the program was 5.3 (range 2–12), while 4.6 (range 1–11) faculty members on average were reported to be directly supervising the trainee in ambulatory clinic. Slightly more than half of the programs (52.2%) responded that their training program was approved by their local hospital graduate medical education office, and a large majority of programs (79.5%) responded that the movement disorders fellow's salary was the same as the corresponding postgraduate year (i.e., PGY-5 or -6) level. There was a substantial variety of funding sources for supporting the fellowship training (Figure 4).

More than half of the programs relied on industry grants (59.1%) or philanthropy other than a Parkinson's disease foundation (52.3%). Other significant sources of funding included institutional support (43.2%), revenue from running clinical trials (34.1%), private donor support (31.8%), and clinical revenue generated by the fellow's movement disorders clinic activities (29.5%). “Other” sources of support included Parkinson Society of Canada grants, VA grants, the Dystonia Medical Research Foundation, faculty NIH grants, and intramural NIH funds.

Fellowship directors were also asked about their fellow's immediate postgraduate employment. The vast majority of fellowship graduates stayed in the US or Canada, with 31.6% in private practice and 63.2% in academic positions.

### 3.5. Current and Future Concerns about Movement Disorders

**Figure 5:**
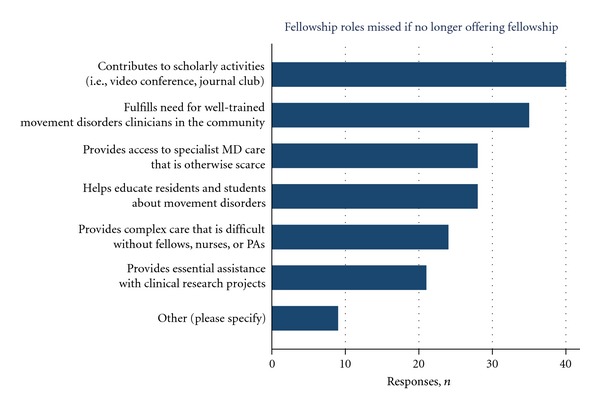
Program directors' opinions regarding the fellow's role within the movement disorders center. Other responses include need for training leaders in clinical research and clinician scientists in the field (*n* = 3).

Fellowship directors were asked about their opinions regarding the benefits of having a movement disorders fellow(s) in their program. Most program directors responded that their presence was important to the center's scholarly activities (Figure 5). The vast majority of programs felt that fellows played important roles in contributing to the group's scholarly activities (90.9%) and fulfilled a need for well-trained movement disorders clinicians in the community (79.5%). Fellows provided access to specialist care that may have been otherwise scarce and valuable teaching assistance for medical students and residents. Notable additional freeform responses by several programs (6.8%) highlighted the critical need for well-trained clinician scientists in movement disorders.

Directors were specifically asked about the current and future need for movement disorders fellowship-trained specialists. Slightly more than half felt that there was currently a noncritical shortage, 54.5%. A critical shortage was noted by 9.1%, while a quarter of respondents felt that there was an adequate supply of trained specialists. In contrast, opinions were mixed about whether the need would be the same in the future, with 31.8% reporting a noncritical shortage, 15.9% reporting a critical shortage, and 36.4% reporting a potentially adequate supply in the future.

**Figure 6:**
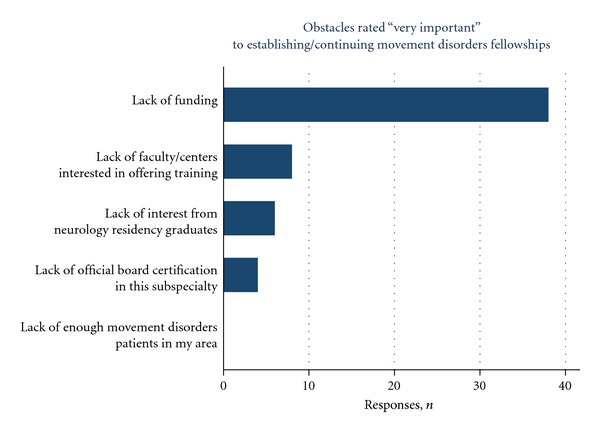
Areas of greatest concern identified by program directors with respect to maintaining fellowship training programs.

Program directors were surveyed regarding their areas of concern with respect to maintaining fellowship training programs. The greatest concern was the lack of funding, with 86.4% responding “very important.” By contrast, there was far less concern about lack of centers and faculty interest in offering training (19.2%), lack of interest among graduating neurology residents (13.6%), or lack of a subspecialty board certifying the specialty (9.1%, Figure 6).

## 4. Discussion

The results of the first multicenter survey of North American movement disorders fellowship training programs highlight several important issues for the field of movement disorders. The response to the survey was excellent among academic centers, especially considering that centers not offering fellowships were unlikely to respond.

North American movement disorders fellowship programs possessed a number of strengths, chief among them being that most centers had major affiliations with leading advocacy and granting organizations. With the support of organizations such as the National Parkinson Foundation, American Parkinson's Disease Association, Parkinson's Disease Foundation, and Veterans Affairs Parkinson's Disease Research Education and Clinical Centers as well as funding through various granting agencies, fellowship programs are often anchored by clinicians and investigators performing leading edge clinical care and research. These centers are uniquely situated to produce properly trained movement disorders specialists to carry on the mission of education, research, and clinical care.

There were broad similarities in the clinical exposure and curriculum content across the different fellowships. Most programs reported between 4 and 6 half-days of ambulatory clinic as the “core” of the clinical experience and also reported dedicated clinics and sessions devoted to DBS intraoperative experience, DBS programming, and botulinum toxin injections. The majority of programs offered regular didactic conferences and meetings, and these were felt to enhance the interaction between trainees and faculty. Many programs offered dedicated clinics in specific diseases or conditions, likely reflecting the unique and diverse expertise of individual programs. Overall, the programs appear to have an abundance of clinical and research material from which trainees could obtain an intensive, high quality training experience at virtually any of the movement disorders fellowship programs surveyed in this study.

The need for producing well-qualified movement disorders clinicians to diagnose, evaluate, and treat these conditions was highlighted by the vast majority of program directors as the most critical role that fellowships serve. Nearly two-thirds of fellowship program directors felt that there was currently at least some shortage of movement disorders specialists, and nearly half felt that this shortage would continue into the future. Additionally, several programs highlighted a concern for maintaining a supply of well-trained clinician scientists.

Nearly a quarter of fellowship programs that responded to the survey did not offer a fellowship slot in the academic year 2011-2012, which was a notable finding. A survey of the past several years revealed that between 16 and 18% of programs did not offer a fellowship slot in each of the preceding years. While this phenomenon could be interpreted as due to the possibility that programs offered two-year fellowships every other year or alternatively did not locate a suitable applicant to fill a vacancy, another potential explanation was the perceived difficulty in locating funding to support a fellowship program on an ongoing basis.

Indeed, in considering chief obstacles to maintaining fellowship programs, the largest concern was a lack of secure funding and funding mechanisms. Nearly 90% of respondents felt that lack of funding was “very important” to the future of movement disorders fellowships. Responders cited a variety of funding sources with considerable reliance on industry grants, private philanthropy, and institutional support and a minority of programs reporting significant support from competitive research training grants from the AAN, NIH, Parkinson Study Group, Dystonia Medical Research Foundation, the VA, and the Parkinson's Society of Canada. Although the number and variety of sources reported were encouraging, since there was overwhelming concern about funding, we surmise that multiple sources of funding may have been a surrogate marker for continued struggles to locate support for fellowship training. We also noted the relative lack of funding granted by the nonprofit disease and research organizations in comparison to private donor and industry support.

Not surprisingly, PD was the most common condition a fellow encountered during a fellowship training experience. PD was estimated to comprise approximately half of all patients seen during the movement disorders fellowship, while tremor disorders, dystonia, and other movement disorders comprised nearly equal proportions of the remaining half. This finding has important implications for the future of clinical care for the growing aging population who may develop PD or related disorders, in addition to the treatment of less commonly seen movement disorders with specific and beneficial therapies. Several studies have observed meaningful differences in specialty care with respect to PD, and more studies are needed to document the importance of movement disorders subspecialty training [2–5]. While more data will be required to demonstrate enhanced outcomes by delivering care through movement disorders subspecialty trained neurologists, this survey identified critical needs in the field. 

We suggest that the movement disorders field needs to prioritize attempts to address the need for sustaining pathways to future advances in research and clinical care. Academic societies, industry parties, government agencies, and foundations may need to work collaboratively to address the funding of movement disorders subspecialty training in order to assure the future health of the field.

## Supplementary Material

The original survey with branching logic items asked of participants depending upon their responses. Programs that did not have fellowship programs were asked for reasons for their not having one and were not asked to fill out questions regarding the structure and content of their training program.
