# Supplementary material for: The Current State and Needs of North American Movement Disorders Fellowship Programs
Source: Parkinsons Dis. 2013 Jul 25;2013:701426. doi: 10.1155/2013/701426 (PMC3745959; doi:10.1155/2013/701426)
Supplement: Supplementary file 1 — The original survey with branching logic items asked of participants depending upon their responses. Programs that did not have fellowship programs were asked for reasons for their not having one and were not asked to fill out questions regarding the structure and content of their training program. [file 701426.f1.pdf]

Movement disorders fellowship program survey

Dear Movement Disorders Center/Fellowship Program Director,

We are conducting an academic research study that aims to assess the status of movement disorders fellowship programs in North America. All programs listed as an NPF Center of Excellence or Care Center or in the AAN Fellowship Directory have been asked to participate. Even if you do not currently offer a fellowship program, we invite you to respond to the survey.

We are asking for one participant from each institution (either fellowship director OR center director, not both) to respond once. Please feel free to forward this link to the appropriate person in your institution. Email reminders will be sent in 4 weeks and 8 weeks if you have not completed the study and would still like to do so.

This survey takes about 10-15 minutes to complete and is completely voluntary. Responses will remain anonymous unless you choose to identify yourself. Answers to questions marked with an asterisk (*) are required before moving to the next page. If you have questions or concerns about this study, please contact Ludy Shih, MD at lshih@bidmc.harvard.edu or 617-667-0519.

Your participation in this study will be part of an important effort to assess the current status and availability of movement disorders fellowship training in North America.

We hope you will complete the survey and add additional comments.

Sincerely,

Ludy Shih

Daniel Tarsy

Michael Okun

Movement disorders fellowship program survey

1. What is the name of your institution/fellowship program?

***2. In which state or province are you located?**

***3. Check all of the following that apply to your center:**

- [ ] Udall Parkinson’s Disease Center
- [ ] VA PADRECC Center
- [ ] NPF Center of Excellence
- [ ] APDA Center of Excellence
- [ ] APDA Information and Referral Center
- [ ] PDF Center of Excellence
- [ ] None of the above
- [ ] Other (please specify)

***4. Have you offered a Parkinson’s disease/movement disorders fellowship in the past?**

- ( ) Yes
- ( ) No

***5. Do you currently offer a Parkinson’s disease/movement disorders (PDMD) fellowship?**

- ( ) Yes
- ( ) No

Reasons for not offering fellowship

***6. If you do not currently offer a Parkinson’s disease/movement disorders fellowship, which of the following reasons apply (check all that apply):**

- ☐ Not enough funds to cover fellow’s salary
- ☐ Not enough clinical volume for optimal training experience
- ☐ Not enough faculty for optimal training experience
- ☐ Too much administrative work
- ☐ Faculty/clinicians not able to donate time to train fellow
- ☐ Training a fellow detracts from the business of clinical care
- ☐ Other (please specify)

Basic program information

***7. In which years have you offered a PDMD fellowship? Indicate how many fellows STARTED that year:**

	0	1	2	3	4	5	>5
2011-12	○	○	○	○	○	○	○
2010-11	○	○	○	○	○	○	○
2009-10	○	○	○	○	○	○	○
2008-9	○	○	○	○	○	○	○
2007-8	○	○	○	○	○	○	○
2006-7	○	○	○	○	○	○	○
2005-6	○	○	○	○	○	○	○
2004-5	○	○	○	○	○	○	○
2003-4	○	○	○	○	○	○	○
2002-3	○	○	○	○	○	○	○
2001-2	○	○	○	○	○	○	○

content of fellowship program

8. Do you offer fellowship slots ONLY to domestic medical graduates (i.e. US medical school graduates for US fellowship programs, Canadian medical school graduates for Canadian fellowship programs)?

- ○ Yes
- ○ No

9. What percentage (0-100) of your clinical fellowship slots have gone to applicants who are international medical graduates?

Number [ ]

***10. What is the typical length of your fellowship program? Choose only one answer.**

- ○ 1 year minimum, no opportunity for 2nd year
- ○ 1 year minimum, 2nd year optional, if institutional funding available
- ○ 1 year minimum, 2nd year optional, if fellow can obtain his/her own external grant funding
- ○ 2 years minimum, funding for both years available
- ○ More than 2 years

11. Do you offer varying lengths of your fellowship program? Check all that apply.

- ☐ 1 year minimum, no opportunity for 2nd year
- ☐ 1 year minimum, 2nd year optional, if institutional funding available
- ☐ 1 year minimum, 2nd year optional, if fellow can obtain grant funding
- ☐ 2 years minimum, funding for both years available
- ☐ More than 2 years

***12. How many half-days each week does the first year fellow work in the following areas?**

	0	<1	1	2	3	4	5	6	7	8	9	10
Outpatient movement disorders clinic	○	○	○	○	○	○	○	○	○	○	○	○
Inpatient consults	○	○	○	○	○	○	○	○	○	○	○	○
Clinical research	○	○	○	○	○	○	○	○	○	○	○	○
Basic research	○	○	○	○	○	○	○	○	○	○	○	○
Teaching/education duties	○	○	○	○	○	○	○	○	○	○	○	○
Didactic classes	○	○	○	○	○	○	○	○	○	○	○	○

13. If you have a second year fellow, how many half-days each week does the second year fellow work in the following areas? If not, please skip question.

	0	<1	1	2	3	4	5	6	7	8	9	10
Outpatient movement disorders clinic	◯	◯	◯	◯	◯	◯	◯	◯	◯	◯	◯	◯
Inpatient consults	◯	◯	◯	◯	◯	◯	◯	◯	◯	◯	◯	◯
Clinical research	◯	◯	◯	◯	◯	◯	◯	◯	◯	◯	◯	◯
Basic research	◯	◯	◯	◯	◯	◯	◯	◯	◯	◯	◯	◯
Teaching/education duties	◯	◯	◯	◯	◯	◯	◯	◯	◯	◯	◯	◯
Didactic classes	◯	◯	◯	◯	◯	◯	◯	◯	◯	◯	◯	◯

14. Of the patients seen by your fellow(s), approximately what percentage have the following conditions? Enter percentages (must add up to 100%).

Parkinson's disease ______

Dystonia ______

Tremor disorders ______

Ataxia/gait disorders ______

Other movement disorders (i.e. chorea, tics, tardive syndromes, various) ______

General neurology (non-movement disorders) ______

***15. Does your fellowship have dedicated ½ or full day clinics for the following specialties? Check all that apply.**

- ☐ Botulinum toxin injections
- ☐ DBS programming
- ☐ DBS intra-operative experience
- ☐ Parkinson’s disease
- ☐ Atypical parkinsonism
- ☐ Dystonia
- ☐ Huntington's disease
- ☐ Tremor
- ☐ Ataxia
- ☐ Tourette and tics
- ☐ Many of above, but interspersed through general movement disorders clinics
- ☐ Other (please specify)

***16. Does your program make efforts to standardize the educational content for each fellow? If so, how? Check all that apply.**

- ☐ Written list of required text and journal article readings
- ☐ Standard schedule of lectures covering specific topics aimed towards the fellow(s)
- ☐ Formal written examinations
- ☐ Formal oral examinations
- ☐ Formal clinical performance examination
- ☐ No, we do not have a formally prescribed curriculum
- ☐ Other (please specify)

***17. Does your fellowship have a regular patient oriented teaching conference (at least monthly)?**

- ☐ DBS conference
- ☐ Video conference
- ☐ Live patient conference
- ☐ Journal club
- ☐ Other (please specify)

***18. Does your fellowship have a regularly occurring didactic lecture based series directed at teaching the basics of movement disorders?**

- ○ Yes
- ○ No

Faculty supervision and accreditation

19. How many movement disorders neurologists are on your fellowship faculty?

Number

20. How many faculty directly supervise your fellow's clinical work in outpatient clinic?

Number

***21. Is your fellowship accredited by your hospital or clinic's local graduate medical education office (GME)?**

- Yes
- No
- I don't know
- N/A

***22. Is the fellow's salary the same, more or less than your hospital's PGY5 or 6 ACGME salaries?**

- Same
- More
- Less
- I don't know
- N/A, no standard ACGME salaries

***23. What sources of funding are used to support the fellow’s salary? Check all that apply.**

- ☐ Industry Grant
- ☐ Philanthropy Other Than a PD Foundation
- ☐ Revenue from running clinical trials
- ☐ Clinical revenue from fellow’s movement disorders clinic activities
- ☐ Clinical revenue from fellow’s general neurology clinic or attending time
- ☐ Salaries of movement disorders faculty
- ☐ Institution support
- ☐ Private donor support
- ☐ APDA fellowship
- ☐ PDF fellowship
- ☐ AAN Clinical Research Training Fellowship
- ☐ NIH T32 training grant
- ☐ APDA Center of Excellence core grant
- ☐ NPF Grants including Center of Excellence
- ☐ Other (please specify)

Fellow's role

***24. If you no longer offered a fellowship, in which of the following ways do you think the movement disorders fellow would be missed? Check all that apply.**

- [ ] Provides patient care and access to specialist MD care that is otherwise scarce
- [ ] Provides essential assistance with clinical research projects
- [ ] Helps educate medical residents and students about movement disorders
- [ ] Contributes to scholarly activities (i.e. video conference, journal club)
- [ ] Provides complex care that is difficult without fellows, nurses, or PAs
- [ ] Fulfills future need for well-trained movement disorders clinician in the community at large
- [ ] Other (please specify)

***25. What positions have your fellows accepted after completion of the fellowship? Enter percentages (must add up to 100%)**

Within US/Canada, private practice	
Within US/Canada, academic position	
Within US/Canada, industry	
Outside of US/Canada, private practice	
Outside of US/Canada, academic position	
Outside of US/Canada, industry	
Another fellowship	
Other	

***26. How likely is your institution to offer a fellowship next year (AY 2012-13)?**

- ○ Unlikely (5% or less)
- ○ Somewhat unlikely (25% or less)
- ○ 50% likely
- ○ Very likely (about 75%)
- ○ Most likely (95% or higher likelihood)

Projected fellowship slots

***27. For AY 2012-13, how many fellowship slots will you offer? Do not count fellows continuing from a prior year.**

Number of slots

Assessing need for movement disorders specialists

***28. Do you think there currently is an adequate number of movement disorders fellowship trained specialists?**

- No, a critical shortage
- No, a shortage, but not critical
- Yes, adequate
- Yes, in excess
- I don't know

***29. Do you think IN THE FUTURE there will be an adequate number of movement disorders fellowship trained specialists?**

- No, a critical shortage
- No, a shortage, but not critical
- Yes, adequate
- Yes, in excess
- I don't know

***30. In considering obstacles to establishing/continuing movement disorders fellowships, rate the following possibilities:**

	Very important	Somewhat important	Neutral	Somewhat unimportant	Very unimportant
Lack of funding					
Lack of faculty/centers interested in offering training					
Lack of interest from neurology residency graduates					
Lack of official board certifying this subspecialty					
Lack of enough movement disorders patients in my area to justify a program					

Other (please specify)

end survey

Thank you for completing the survey.

31. Comments:
